# Application of Virtual Reality in Spatial Memory

**DOI:** 10.3390/brainsci13121621

**Published:** 2023-11-23

**Authors:** José Manuel Cimadevilla, Raffaella Nori, Laura Piccardi

**Affiliations:** 1 Department of Psychology, University of Almeria, 04120 Almeria, Spain; jcimadev@ual.es; 2 Health Research Center, University of Almeria, 04120 Almeria, Spain; 3 Department of Psychology, University of Bologna, 40127 Bologna, Italy; raffaella.nori@unibo.it; 4 Department of Psychology, Sapienza University of Rome, 00185 Rome, Italy; 5 San Raffaele Cassino Hospital, 03043 Cassino, Italy

In recent years, virtual reality (VR) has become a widely used tool with a plethora of applications in neuroscience. In particular, VR has the potential to greatly enhance spatial memory in a wide range of applications. For example, it can be used to simulate real-world environments, becoming particularly valuable in training individuals with navigation difficulties—such as those with neurodegenerative disorders, acquired brain damage, neurodevelopmental disorders (i.e., developmental topographical disorientation), or psychological diseases, such as spatial anxiety, agoraphobia or visual impairments—helping them navigate their surroundings with greater confidence (e.g., [1,2]). Additionally, studies have shown that VR can recreate complex spatial layouts, allowing users to practice their wayfinding skills and improve their spatial memory (e.g., [3,4]). VR is also used to enhance spatial memory and learning in educational settings. Subjects like geography, history, and anatomy benefit from a VR experience that transports students to different locations and periods, facilitating the retention of spatial information (e.g., [5,6]).

Furthermore, VR allows for interactive learning experiences, where students can manipulate and explore spatially complex concepts, leading to deeper understanding and improved spatial memory. Being an engaging device that strengthens spatial memory and mental mapping abilities through gaming, VR also represents an invaluable tool for children with specific learning disabilities [7,8,9]. Overall, the utilization of VR in enhancing spatial memory has extensive benefits across various fields, including healthcare, architecture, education, and entertainment. By immersing users in realistic virtual environments, VR improves spatial memory and cognitive skills, ultimately enhancing the user’s ability to navigate, learn, and interact with the world around them. This Special Issue is devoted to gathering studies on VR and spatial memory. It includes eight contributions covering the application of VR for both children and adults. Six studies investigate which factors affect spatial navigation in VR: two investigate these factors during developmental ages (i.e., [10,11]), and the other four focus on young adults (i.e., [12,13,14,15]). One study reviews the applications of radial arm maze in both virtual and real versions (i.e., [16]), and the study by Zucchelli et al. [17] shows how VR can be a proper differential diagnosis tool for mild agoraphobia disorders. The latter study highlights, through the use of VR, the presence of deficits in visuospatial working memory and navigation in people with agoraphobic-type anxiety disorders.

More specifically, the first contribution by Bocchi and co-workers [12] investigated individual factors like gender and familiarity and their effect on environmental representation acquired during spatial virtual navigation (Almeria Boxes Tasks). These authors found a difference between the sexes, with men seeming to prefer relying on map-like survey representations, while women prefer using sequential route representations. They also found that the weight of familiarity with the environment favors a complete mental environmental representation. This last finding was in line with evidence that familiarity can mitigate gender differences in spatial tasks, especially in more complex ones. By using the same virtual environment with different spatial memory tasks resulting in different cognitive demands (Almeria boxes tasks), Tascón and co-workers [13] investigated how men and women perform and concluded that familiarity with the spatial context determined sex differences, the difficulty level of the task, the active or passive role of the participant, and the amount of visual information provided in each screenshot. Also, in this contribution, the uniqueness of VR emerges, allowing for minimal variations of the same environment, enabling a high degree of experimental control and, consequently, conclusions free of disturbing variables. Meneghetti and Pazzaglia [14] also utilized a virtual environment to explore the factors that enhance spatial learning. They examined the advantages of viewing a map before navigation, reading verbal descriptions of the environment, and navigating without any prior information. Their findings indicated that viewing a map beforehand improved learning accuracy. Additionally, recall performance was partially influenced by individual visuospatial and verbal abilities.

van Dunn and colleagues [10] focused instead on other factors that might affect performance in a virtual environment. Mainly, they investigated the gaming experience in children 9 to 11 years of age, taking into account that girls generally play video games less than boys and that this factor could have a bearing in determining the gender differences in adulthood that also emerge in the two studies mentioned above ([12,13]). The experience of being a video gamer could result in the person acquiring familiarity with the environment more quickly, thus requiring less repetition or shorter exposure to the environment. Van Dunn et al. [10] found that boys navigated faster than girls, and this difference increased with age. Ultimately, boys having more gaming experience than girls did not explain any of the observed results. This seems to contrast with the literature, which has established a reasonably robust correlation between practice with videogames and enhancement of visuospatial skills and spatial navigation (e.g., [18,19,20]). However, Milani et al. [21] clarified that the positive effects on spatial competencies are related to the type of visuospatial skill measured. It is also possible that this effect could be age-related, explaining why von Dun et al. [10] did not find differences between girls and boys despite boys playing more video games than girls.

Staying in the developmental field, van Hoogmoed et al. [11] conducted a study on the development of landmark-based navigation in children 5 to 10 years of age. Using a non-immersive virtual environment, they investigated the developmental trajectories of egocentric and allocentric perspectives. They found that allocentric navigation started to develop between 5 and 8 years of age and was related to sex, with boys outperforming girls. Both boys and girls relied more on directional than positional landmark information.

Continuing to focus on factors that can influence performance in a virtual environment, Miola et al. [15] conducted a study on young adults to explore the impact of self-efficacy on learning in a virtual environment. They divided the sample into two groups—one receiving positive feedback and the other neutral feedback. The study found that receiving positive feedback after completing a visuospatial task improves environmental recall. Additionally, the results suggest that task-specific self-efficacy is crucial in sustaining environmental learning among individuals.

Also seeking to compare virtual and natural settings, Palombi et al. [16] write a review highlighting how the radial arm maze (RAM) is a paradigm used to study spatial abilities in real and virtual environments. In recent years, the use of VR-RAM has increased, also as a neuro-rehabilitation tool. Undoubtedly, new research is needed to better understand the role these tools can play in assessing and rehabilitating patients, something that has gained in importance in the pandemic era when alternative and remote methods have increasingly been sought.

In contrast, the contribution by Zucchelli, Piccardi, and Nori [17] concerns the use of VR in treating the anxiety disorder agoraphobia, highlighting valuable findings for the differential diagnosis of the disorder even in its mild form. These authors used a virtual environment to study spatial memory in individuals with agoraphobia. They found that these individuals, although not suffering from a severe and disabling form of agoraphobia, had a diminished visuospatial working memory and that, as the number of individuals in the virtual environment increased, they became less able to make egocentric and allocentric judgments of the environment. The impairment of visuospatial working memory would appear to mediate spatial orientation in such individuals. Due to the challenges faced by those suffering with agoraphobia in using public transportation, being in enclosed or open spaces, or being in crowded areas [22], conducting a study in a real-life setting would have been impossible. However, VR provides a way for clinicians to assess and treat people with this disorder, who may otherwise struggle with being in public spaces or going outside in general. VR is an excellent tool for clinicians to help those with agoraphobia manage their anxiety and enhance their overall quality of life with the proper support and treatment.
